# (Pro)Renin receptor mediates obesity-induced antinatriuresis and elevated blood pressure via upregulation of the renal epithelial sodium channel

**DOI:** 10.1371/journal.pone.0202419

**Published:** 2018-08-17

**Authors:** Syed S. Quadri, Silas Culver, Nrupama Ramkumar, Donald E. Kohan, Helmy M. Siragy

**Affiliations:** 1 DeBusk College of Osteopathic Medicine, Lincoln Memorial University, Harrogate, Tennessee, United States of America; 2 Department of Medicine, University of Virginia Health System, Charlottesville, Virginia, United States of America; 3 Division of Nephrology and Hypertension, University of Utah Health Sciences Center, Salt Lake City, Utah, United States of America; Max Delbruck Centrum fur Molekulare Medizin Berlin Buch, GERMANY

## Abstract

Recent studies have demonstrated that the renal (pro)renin receptor (PRR) regulates expression of the alpha subunit of the epithelial sodium channel (α-ENaC). In this study we hypothesized that the renal PRR mediates high fat diet (HFD)-induced sodium retention and elevated systolic blood pressure (SBP) by enhancing expression of the epithelial sodium channel (α-ENaC). In our study we used a recently developed inducible nephron specific PRR knockout mouse. Mice (n = 6 each group) were allocated to receive regular diet (RD, 12 kcal% fat) or a high-fat diet (HFD, 45 kcal% fat) for 10 weeks. Body weight (BW), SBP, urine volume (UV) and urine sodium (U_Na_V), as well as renal interstitial Angiotensin II (Ang II), and renal medullary expression of PRR, p-SGK-1, α-ENaC were monitored in RD and HFD mice with or without PRR knockout. At baseline, there were no significant differences in BW, BP, UV or U_Na_V between different animal groups. At the end of the study, HFD mice had significant increases in SBP, BW, and significant reductions in UV and U_Na_V. Compared to RD, HFD significantly increased mRNA and protein expression of PRR, α-ENaC, p-SGK-1, and Ang II. Compared to HFD alone, PRR knockout mice on HFD had reduced mRNA and protein expression of PRR, p-SGK-1, and α-ENaC, as well as increased UV, U_Na_V and significantly reduced SBP. RIF Ang II was significantly increased by HFD and did not change in response to PRR knockout. We conclude that obesity induced sodium retention and elevated SBP are mediated by the PRR-SGK-1- α-ENaC pathway independent of Ang II.

## Introduction

Obesity is a national and global epidemic which puts the individual at higher risk for heart disease, stroke, diabetes, and high blood pressure (BP). As much as 75% of essential hypertension is attributable to increased body weight [1] and the fact that reductions in body weight significantly decrease blood pressure supports this causal relationship.[2–4] However, the underlying mechanisms governing the relationship between obesity and the increased sodium retention leading to elevated BP are incompletely understood.

One of the known causes of elevated BP during obesity is impaired renal natriuresis which increases renal sodium retention in obesity.[5] Other factors contributing to obesity induced sodium retention are increased sympathetic nervous system activity, increased renin-angiotensin-aldosterone system (RAS) activity, decreased levels of natriuretic peptides, fat compression of the kidney, and an increase in inflammatory cytokines.[6,7] However, our understanding of all the factors involved is far from comprehensive. While obesity increases the sodium retention in various parts of the nephron,[5] the precise mechanisms regulating these changes have not been fully explained.

The amiloride-sensitive epithelial sodium channel (ENaC) is an important transporter that plays a critical role in maintaining Na^+^ homoeostasis by enhancing its absorption.[8] ENaC is comprised of three subunits, α, β, and γ;[9] which are highly expressed from the distal convoluted tubule to the collecting duct in the nephron.[10,11] Of the three subunits, α-ENaC is critical for channel activity [9] while the β- and γ- ENaC subunits play a complementary role to the function of α-ENaC.[9] In obesity, upregulation of ENaC is associated with increased sodium retention contributing to the development of increased BP [12,13] and we recently demonstrated that decreased renal α-ENaC expression increases urinary sodium excretion.[14] These findings highlight the importance of understanding the regulation of α-ENaC in obesity-induced hypertension.

The RAS plays an essential role in the regulation of blood pressure, renal hemodynamic and tubular sodium reabsorption. (Pro)renin receptor (PRR) is a component of the RAS which binds to renin and prorenin and enhances their catalytic activity.[15] PRR is a single transmembrane protein localized mainly in renal vasculature, proximal and distal tubules, and collecting ducts.[9,15,16] Activation of PRR stimulates a variety of signal transduction pathways including ERK and mitogen-activated protein kinase.[17,18] While PRR was initially described as a component of the RAS, its RAS independent role in sodium handling via α-ENaC has also been recently demonstrated.[14]

To date no studies have investigated the significance of PRR in obesity induced antinatriuresis. Based on current knowledge we hypothesize that, in high fat diet (HFD) induced obesity, PRR promotes increased renal sodium retention and higher BP via upregulation of the α-ENaC sodium channel. In the present study we used a recently developed inducible nephron-wide knockout model of PRR to test our hypothesis.[19]

## Materials and methods

### Animal preparation

The University of Virginia Animal Care and Use Committee approved all study protocols. Mice were allowed one week to adjust to our animal care facility and were provided tap water ad libitum and normal sodium diet (Harlan-Teklad, Madison, WI). Ketamine/ Xylazine were used for anesthesia during collection of renal interstitial fluid, organ harvesting and euthanasia.

#### Induction of Nephron-specific PRR KO

In the present study we used recently developed nephron-specific inducible PRR KO mice which were generated as described earlier.[19] These mice were kindly provided by Drs. Donald E. Kohan and Nirupama Ramkumar, University of Utah Health Sciences Center, Salt Lake City, Utah. Briefly, these mice are bred on a C57BL/6J background and are hemizygous for Pax8-rTA and LC1 and are homozygous for a floxed PRR gene. Nephron specific PRR knockout is induced by administration of doxycycline in drinking water as described below. Both male and female mice were used in these studies. Starting at 10 weeks of age, nephron specific PRR KO mice (n = 6) were provided with tap water ad libitum and were either fed regular diet (RD, 12% fat, 0.4% sodium chloride) (Harlan-Teklad) or high fat diet (HFD, 45% fat from lard, 0.3%sodium chloride) (Research Diets) for a total of 10 weeks. To induce nephron-wide PRR KO, after the initial 4 weeks of diet treatment, mice were treated with 2 mg/ml doxycycline in 2% sucrose drinking water for 12 days while control mice (those not undergoing PRR knockout) received 2% sucrose water only for the same period. All mice were then placed back on normal tap water for the remainder of the 10 week diet treatment.

### Genotyping

DNA was extracted from mouse tails and PCR was performed using the following primers:

PAX-8-rtTA (600 bp), forward: 5’-CCATGTCTAGACTGGACA AGA-3’; reverse: 5’-CTCCAGGCC ACATATGAT TAG-3’.

PRR (600 bp), forward: 5′- GGGGGGTAAATTGTTGATGAGTCTTGGAGCATAGC-3′; reverse 5′-GAAGCCCATGGACAGTGCAGCTACGTCTGGGATTCGA-3′.

LC-1 (480 bp), forward: 5′- TCGCTGCATTACCGGTCGATGC-3′; reverse 5′-CCATGAGTGAACGAACCTGGTCG-3’.

#### Systolic blood pressure, body weight, food intake, 24 hour urine

BP was assessed on day 0 (baseline) and week 10 post diet as previously described[20] in non-anesthetized mice using a tail-cuff non-invasive multi channel blood pressure system (IITC Life Sciences, Woodland Hills, CA). Briefly, mice were placed in a chamber on a heating pad at 37°C for 10 min and transferred to tail-cuff blood pressure system (IITC Life Sciences, Woodland Hills, CA). The tail cuff was connected to a cylinder of compressed air through an arrangement of inlet and outlet valves that permitted inflation and deflation of the cuff at a constant rate. The tail-cuff pressure was continuously recorded. The signals from the pulse and pressure sensors were amplified and then digitized with an analog–digital board mounted in a desktop computer. All mice were acclimatized to the blood pressure device prior to taking tail cuff measurements. Body weights, food intake and 24 hour urine collections were made using individual metabolic cages. The total urine volume was determined and urine aliquots were stored at -80°C until assayed.

#### RT-PCR analysis

Quantitative real-time reverse transcriptase- polymerase chain reaction (RT-PCR) was used to determine changes in renal medullary mRNA expressions of PRR and ENaC. The RNA (n = 4 each group) was extracted using Trizol (Invitrogen, Carlsbad, CA). Reverse transcription of the RNA was performed using a first strand cDNA synthesis kit (Bio-Rad, Hercules, CA). The PCR was analyzed using SYBR Green Supermix (Bio-Rad). Mouse Primer sequences were as follows: PRR, forward sequence 5’-TCTCCGAACTGCAAGTGCTA-3’; reverse sequence 5’-CTGCAAACTTTTGGAGAGCA-3’; α –ENaC, forward sequence 5′-CTAATGATGCTGGACCACACC-3’; reverse sequence 5’-AAAGCGTCTGCTCCGTGATGC-3’; β-actin, forward sequence 5’-AGCCATGTACGTAGCCATCC-3’; reverse sequence 5’-ACCCTCATAGATGGGCACAG-3’. Reactions were performed in triplicate and threshold cycle numbers were averaged. The mRNA results for specific target genes were calculated with normalization to β-actin mRNA.

#### Western blot

Antibodies to PRR (1:1000 dilutions, Abcam, Cambridge, MA, USA), p-SGK-1 (1:1000 dilutions, Cell signaling, USA), SGK-1 (1:1000 dilutions, Cell signaling, USA), α-ENaC (1:500 dilutions; ASC-030, Alamone labs, Israel) were used in the Western blot of renal medullary protein as previously described.[20,21] Protein expressions were normalized to β-actin protein (1:1000 dilutions, Santa Cruz, Dallas, TX, USA) Immunoblotting was done using an n of 4 for all treatment groups.

#### In vivo renal interstitial fluid (RIF) collections and assays for Ang II

In vivo RIF for Ang II measurements was collected by microdialysis at the end of the study as previously described.[22] Ang II concentrations were determined using a commercially available ELISA kit (Cayman Chemical, Ann Arbor, MI, USA).[23]

#### Statistical analysis

Comparisons among different treatment groups were assessed by Student *t* test when appropriate or by one- way ANOVA followed by a Tukey test for post-hoc comparisons. Data were expressed as mean ± SE. P<0.05 was considered statistically significant.

## Results

### Systolic blood pressure, Body weight, 24 hour food intake

**Fig 1:**
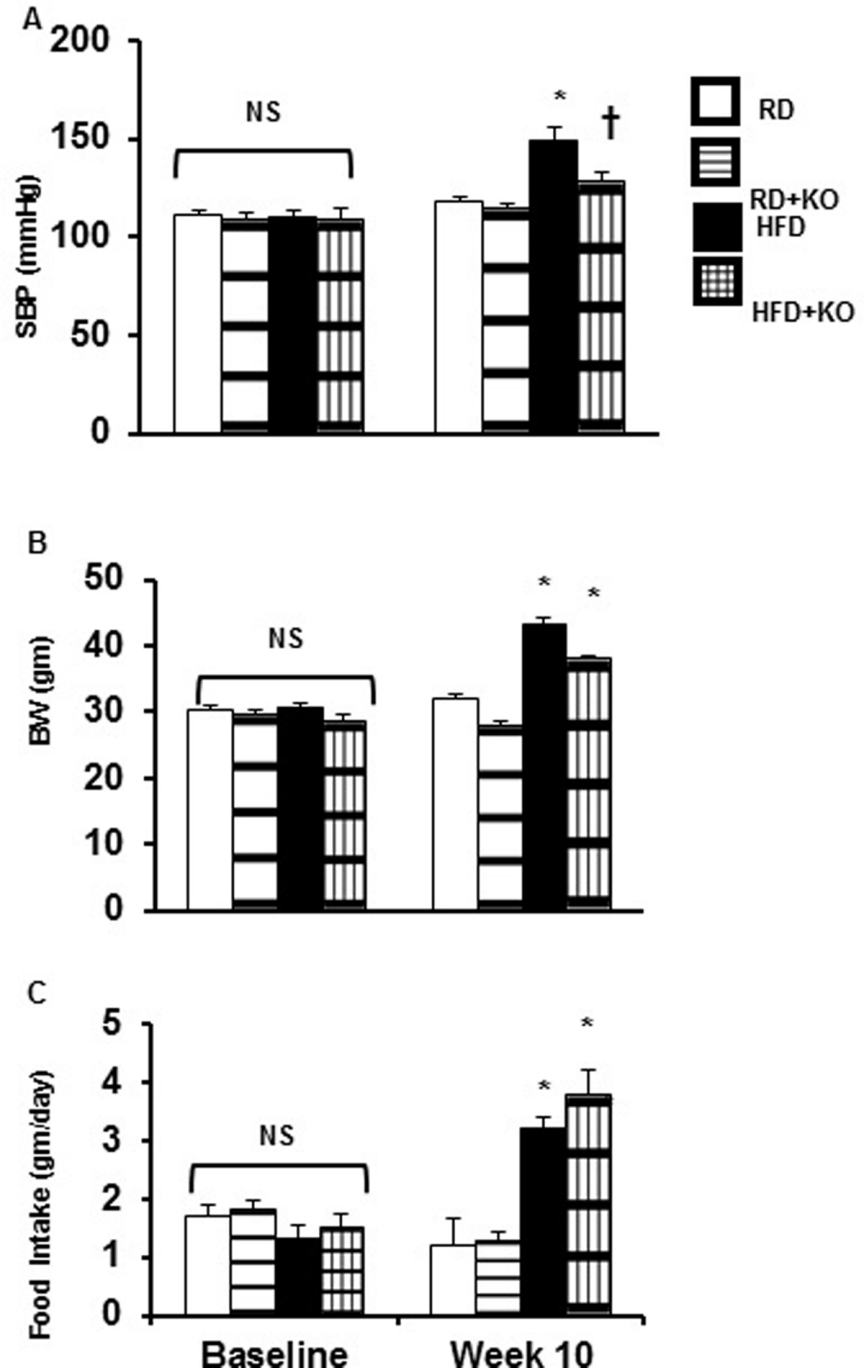
Systolic blood pressure, body weight and 24 hour food intake. (A) Systolic blood pressure (SBP), (B) Body Weight (BW), and (C) 24 Hour Food Intake at baseline and after 10 weeks following regular diet (RD) and high fat diet (HFD) in mice with or without PRR KO. Data presented as mean ± SEM, NS no significant difference, *p<0.05 vs RD and †p<0.05 vs HFD, n = 6 each group.

Compared to RD control mice (RD) (10 weeks, 12 kcal% fat), control mice fed HFD (HFD) (10 weeks, 45 kcal% fat) had a significant increase in systolic blood pressure (SBP) (HFD 149.4 ± 6.04 mmHg, vs. RD 111.4 ± 2.54 mmHg, P<0.05) (Fig 1A), BW (HFD 43.2 ± 1.125 gm, vs. RD 31.9 ± 0.89654 gm, P<0.05) (Fig 1B) and 24 hour food intake (RD 1.2 ± 0.48 gm, vs. HFD 3.2 ± 0.21 gm, P<0.05) (Fig 1C). Compared to HFD controls, PRR KO induction (HFD + PRR KO) significantly reduced the SBP (HFD 149.4 ± 6.04 mmHg vs. HFD + PRR KO 129 ± 3.75 mmHg P<0.05) (Fig 1A). PRR KO did not change BW (HFD 43.2 ± 1.125 gm, vs. HFD + PRR KO 38.0 ± 0.36 gm) (Fig 1B) or 24 hour food intake (HFD 3.2 ± 0.21 gm vs. HFD + PRR KO 3.8 ± 0.42 gm) (Fig 1C). There were no significant differences in BP, BW and 24-h food intake at baseline (day 0) (Fig 1).

### Urinary volume (UV) and Urinary sodium excretion (U_Na_V)

**Fig 2:**
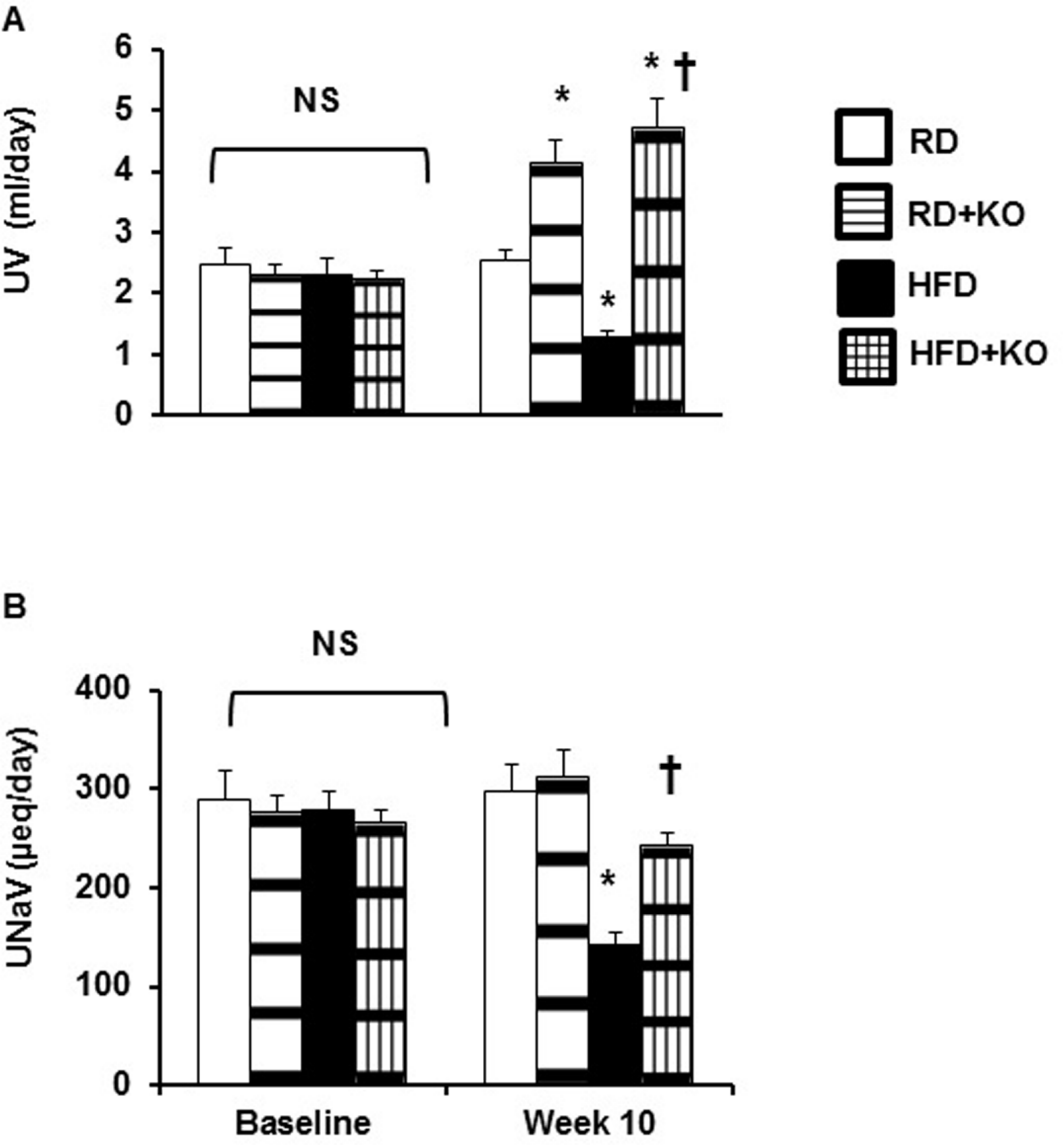
Urine volume and urinary sodium excretion. (A) Urine Volume (UV) and (B) Urinary Sodium Excretion (U_Na_V) at baseline and after 10 weeks following regular diet (RD) and high fat diet (HFD) in mice with or without PRR KO. Data presented as mean ± SEM, NS no significant difference, *p<0.05 vs RD and †p<0.05 vs HFD, n = 6 each group.

Given the differences in SBP between different treatment groups, we examined whether there was evidence for changes in renal sodium excretion. There were no significant differences in UV and U_Na_V at baseline (day 0) between different treatment groups (Fig 2A and 2B). At the end of 10 weeks, compared to RD controls, control mice fed HFD (HFD 1.3 ± 0.08 ml/day, vs. RD 2.54 ± 0.18 ml/day, P<0.05) had a significant decrease in the 24 hour UV (Fig 2A). Compared to RD controls, RD + PRR KO significantly increased the UV by 62% (RD + PRR KO 4.12 ± 0.37 ml/day vs RD 2.54 ± 0.18 ml/day, P<0.05). Compared to HFD alone, HFD + PRR KO increased the UV by 263% (HFD + PRR KO 4.72 ± 0.42 ml/day, vs. HFD 1.3 ± 0.08 ml/day, P<0.05) (Fig 2A).

Similarly, compared to RD, mice fed with HFD (HFD 141.38 ± 13.3 μEq/day vs. RD 297.05 ± 26.8 μEq/day, P<0.05) had a significant decrease in the 24 hour U_Na_V (Fig 2B). Compared to HFD alone, HFD + PRR KO increased the U_Na_V by 41% (HFD + PRR KO 242.6± 6.35 μEq/day, vs. HFD 141.38 ± 13.3 μEq/day, P<0.05) (Fig 2B). There were no changes in U_Na_V between RD and RD + PRR KO.

### Expression of PRR in response to HFD

**Fig 3:**
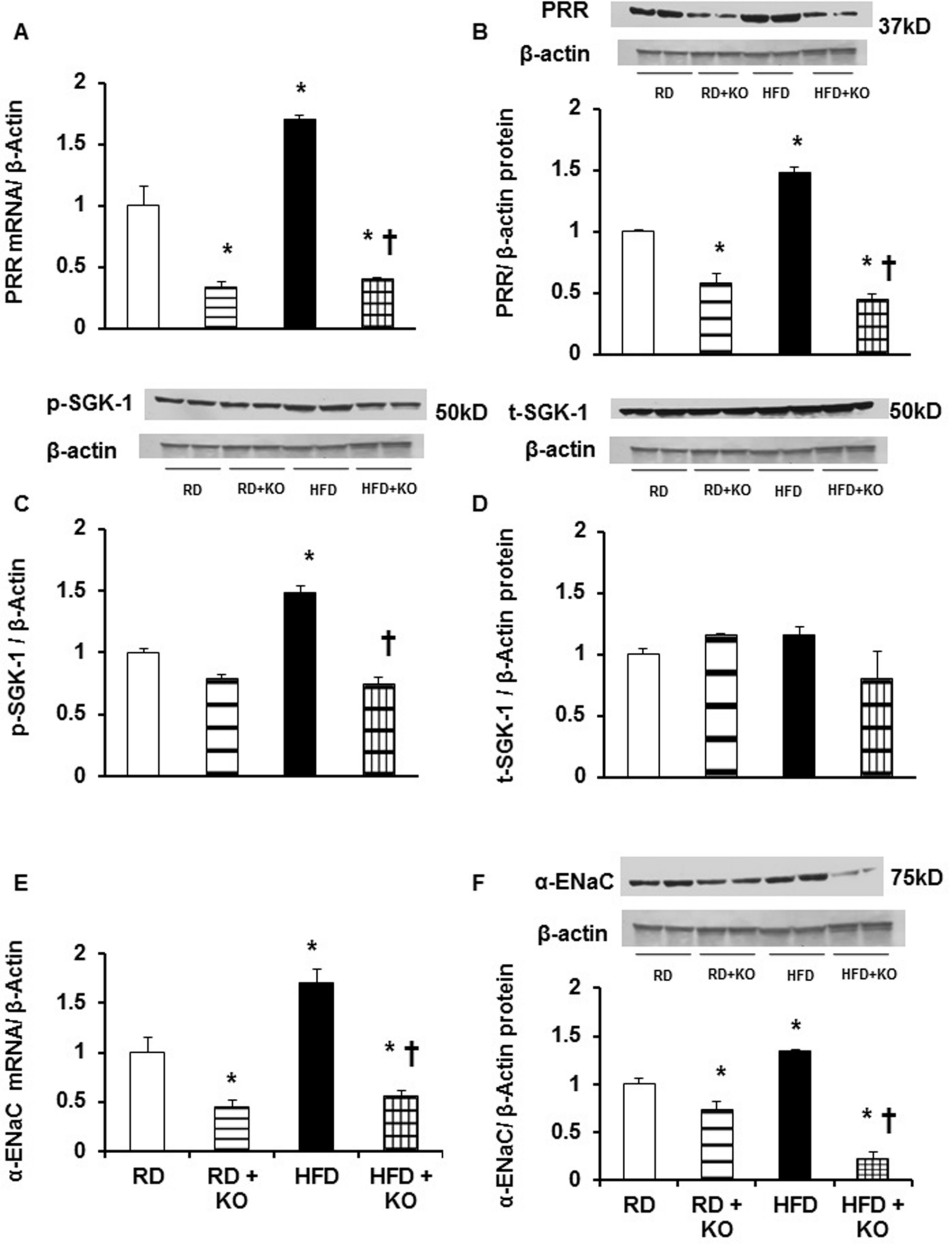
Renal medullary expression of PRR, SGK-1, and α-ENaC. Renal medullary mRNA (A) and protein (B) expressions of (Pro)renin receptor (PRR), phosphorylation of SGK-1 (C) and total expression of SGK-1 (D), mRNA (E) and protein (F) expressions of α-ENaC in mice fed with regular diet (RD) and high fed diet (HFD) with or without PRR KO. mRNA normalized to β-actin, representative blots (top), quantitative results normalized to β-actin. Data presented as mean ± SEM, NS no significant difference, *p<0.05 vs RD and †p<0.05 vs HFD, n = 4 each group.

Renal medullary PRR mRNA and protein expression is shown in Fig 3A and 3B. Compared to RD, renal medullary mRNA and protein expression of PRR was significantly increased in mice fed with HFD by 69% and 47% respectively (P<0.01). Compared to RD alone, RD + PRR KO significantly decreased the mRNA and protein expression of PPR by 67% and 43% (P<0.05). Compared to HFD alone, HFD + PRR KO significantly decreased the mRNA and protein expression of PPR by 76% and 70% (P<0.001).

### Effect of PRR KO on p-SGK-1 and α-ENaC expression

SGK-1 is responsible for regulating the expression of α-ENaC in the distal nephron [24] and we therefore measured changes in SGK-1 and α-ENaC expression with HFD and PRR KO. Compared to RD, there were significant increases in mRNA and protein expression of α-ENaC by 70% and 35% respectively (P<0.05)(Fig 3E and 3F) and phosphorylation of p-SGK-1 by 48% (P<0.05) (Fig 3C) in mice fed with HFD. Compared to RD alone, RD + PRR KO significantly attenuated the renal mRNA and protein expressions of α-ENaC by 55% and 27% respectively (P<0.05) (Fig 3E and 3F). Compared to HFD alone, HFD + PRR KO mice also significantly attenuated the renal mRNA and protein expressions of α-ENaC by 67% and 83% respectively (P<0.05)(Fig 3E and 3F) and p-SGK-1 by 50%, (P<0.01)(Fig 3C). There were no changes in total SGK-1 expression (Fig 3D).

### Renal interstitial fluid angiotensin II (Ang II)

**Fig 4:**
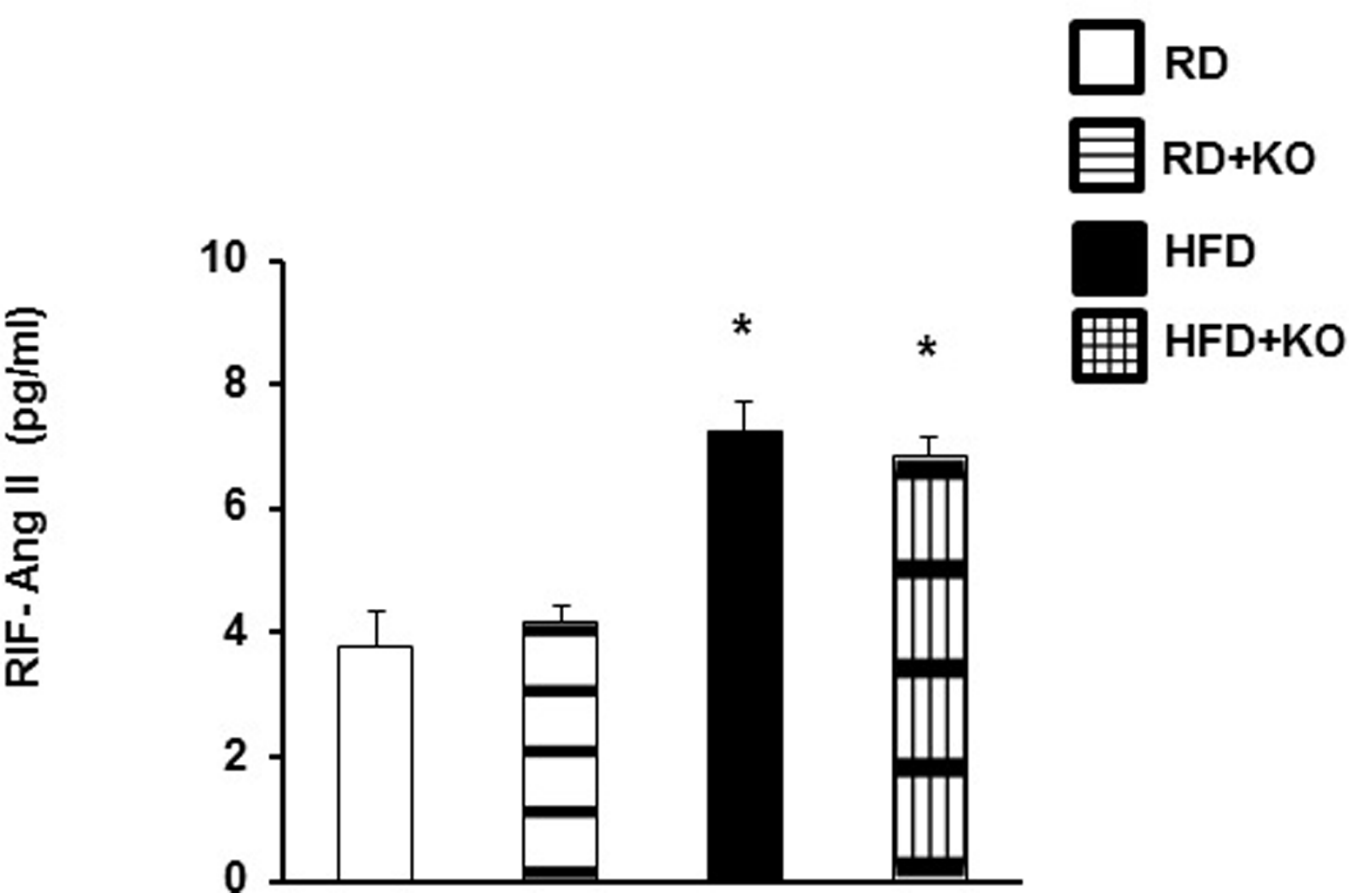
RIF Ang II levels. Renal interstitial fluid (RIF) expression of angiotensin II in kidneys of RD and HFD mice with or without PRR KO. Data presented as mean ± SEM, *p<0.05 vs RD n = 4 each group.

As Ang II can also stimulate α-ENaC expression and activity [25,26], we measured Ang II levels in RIF to determine whether the observed changes in α-ENaC and blood pressure with PRR knockout were Ang II independent. Compared to RD, HFD increased RIF Ang II by 92% (RD 3.75 ± 0.5 pg/mL vs. HFD 7.2 ± 0.47 pg/ml, P<0.001) (Fig 4). Nephron specific PRR KO in both RD and HFD did not change RIF Ang II levels. (Fig 4).

## Discussion

Multiple factors contribute to obesity related sodium retention and elevated BP, including increased sympathetic nervous system activity, increased RAS activity, decreased levels of natriuretic peptides, compression of the kidney by excess adipose tissue, and an increase in inflammation.[5,27] The common element in all of these processes, however, is impaired natriuresis.[5] Therefore, elucidating the regulation of renal sodium channels in obesity is key to enhancing our understanding of this highly prevalent and morbid condition.

The present study demonstrates that renal PRR promotes obesity induced increases in blood pressure by increasing expression of α-ENaC, leading to increased renal sodium retention. This result is consistent with recent data from our laboratory showing that renal PRR is capable of regulating α-ENaC expression through the SGK-1-Nedd-4-2-α-ENaC pathway. [14] Similarly, inner medullary collecting duct cells treated with low salt medium had increased α-ENaC expression that was reversed by PRR shRNA.[14] Recent work by Ramkumar et al using the same nephron specific PRR knockout model as in the current study, also demonstrated that under baseline conditions knockout of PRR in the nephron resulted in decreased α-ENaC expression.[28] To our knowledge, however, the current study is the first to show increased renal PRR in the setting of obesity and that this in turn mediates obesity related increases in BP via α-ENaC.

Importantly, this study also demonstrates that in obesity, renal PRR regulates α-ENaC independent of the RAS. PRR is known to signal through both Ang II dependent and independent pathways.[18,29] At the same time, Ang II itself has previously been shown to increase expression of both PRR and α-ENaC in the distal nephron.[26,30] Our results, however show that while renal Ang II did increase in obese mice, Ang II levels were unchanged by PRR knockout while α-ENaC and sodium retention decreased in the absence of nephron PRR. This finding is consistent with our previous data in low salt treated inner medullary collecting duct cells where PRR also upregulated α-ENaC independent of Ang II.[14] In addition, recent evidence indicates that PRR may in fact mediate the effects of Ang II on α-ENaC.[28] This may further explain how PRR knockout was able to significantly increase renal sodium excretion despite the continued elevation in renal ang II. Another recent study using collecting duct specific PRR knockout mice reported a blunted blood pressure response to Ang II infusion in conjunction with reduced expression of cleaved α-ENaC and lower ENaC channel activity.[31] While significant differences also exist between these two models (the collecting duct specific knockout mice had lower glomerular filtration rate and blood pressure at baseline compared to controls while the nephron specific model did not), they reinforce the idea that PRR in the nephron mediates Ang II effects on renal sodium handling while PRR itself is capable of signaling independent of RAS.

Our study does leave several unanswered questions. While we convincingly show that obesity upregulates PRR with subsequent increases in α-ENaC, the precise mechanism by which PRR does this remains unclear. It is well established that the PI3K/Akt/mTOR pathway is capable of activating SGK-1, the primary regulator of α-ENaC.[32,33] We have also previously shown that PRR is capable of activating the PI3K/Akt/mTOR pathway under high glucose conditions.[34] In the previously mentioned study by Ramkumar et al, prorenin stimulated α-ENaC expression in a PRR dependent manner and this was prevented by inhibition of Akt.[28] While further studies are clearly needed to specifically investigate how PRR mediates an increase in α-ENaC in obesity, these findings strongly suggest that PI3K/Akt/mTOR could be involved in this pathway.

It also remains unclear what promotes increased renal PRR expression in the setting of obesity. PRR expression is upregulated in a number of models of renal disease including diabetic nephropathy, Goldblatt hypertension, and chronic kidney disease.[16,22,23,35] We previously showed that the pro-inflammatory factor NF-κB increases PRR expression under high glucose conditions by binding to the PRR promoter.[16] This indicates that inflammatory mediators can act as a positive regulators of PRR expression and it is also well accepted that obesity is a condition of increased inflammation.[5,36,37] While it is possible that inflammation present in obesity promotes renal PRR expression, our current study did not measure markers of systemic or renal inflammation. It could also be speculated that other factors such as RAS or leptin, which are known to be upregulated in obesity, could be acting upstream of PRR but these pathways were also not part of our current study.

In addition to α-ENaC, other renal sodium transporters are known to be involved in mediating obesity related hypertension[38] and future studies should address the role of PRR in regulating these transporters and their activity in obesity. Specifically, our study does not quantify the expression or activity of these other channels nor does it employ channel specific inhibitory agents to demonstrate the specific effect of one channel over another. We nonetheless believe that there is sufficient prior evidence to support the concept that α-ENaC is the major mediator for PRR regulated sodium transport in obesity. For one, it has already been established that hyperactivity of the renal sodium channel results in salt-sensitive hypertension in both humans and rodent models.[39] Secondly, previous studies on the role of renal tubular epithelial cell PRR in non-obese models of hypertension also demonstrated that knockout of PRR reduced expression of α-ENaC and prevented hypertension without changes in expression of the NHE3, NKCC2, or NCC transporters.[28] While it thus remains to be seen whether PRR contributes to obesity induced BP elevation through effects on additional sodium transporters, it is reasonable to conclude that α-ENaC is central to this process.

In terms of limitations, this study admittedly did not measure changes in renal function in response to nephron specific PRR knockout. While this knockout model does have decreased urine concentrating ability, prior studies of this model have shown that under baseline conditions, the knockout does not change BUN or urine sodium and potassium.[19] Our findings regarding urine sodium in this study under RD conditions are thus consistent with these earlier results. It should also be acknowledged that the dietary sodium content of the high fat diet used in this study was slightly lower than that of the regular diet. However these differences were quite small and do not explain the fact that PRR knockout in the nephron significantly increased urine sodium excretion under HFD conditions. Rather this result would suggest that a significant degree of the sodium retention observed in HFD mice was dependent on PRR.

This study also has several strengths. Use of a recently developed inducible nephron specific PRR knockout model is preferable to the use of shRNA as it permits specific targeting of the structures directly involved in renal sodium handling. It is also superior to constitutive knockout of the gene in that it allows for normal development and organogenesis prior to induction. Prior characterization of the model used in this study showed it to have normal renal histological development and normal survival.[19] This is especially pertinent as PRR has previously been implicated in renal embryogenesis[40] and because obesity is a condition that usually develops over time during the life of the organism.

In conclusion, this study provides evidence that renal PRR plays a significant role in the pathogenesis of obesity induced renal sodium retention and BP elevation through upregulation of α-ENaC in an Ang II independent manner. Based on the current study and our previous reports[14], we propose that sodium retention and BP elevation associated with obesity are mediated by PRR-SGK-1-Nedd-4-2 ubiquitination/expression of α-ENaC (Fig 4). Our findings have significant therapeutic implications for the treatment of hypertension in obesity as they potentially represent a novel target for regulating renal sodium handling beyond previously established mechanisms. Obesity induced antinatriuresis and elevated BP is nonetheless a complex disease state and further study is needed to better understand how the relationship between PRR and α-ENaC fits into the broader scope of renal changes that occur in obesity.
